# Effects of the Application of Digestates from Wet and Dry Anaerobic Fermentation to Japanese Paddy and Upland Soils on Short-Term Nitrification

**DOI:** 10.1264/jsme2.ME14080

**Published:** 2015-02-04

**Authors:** Kozue Sawada, Koki Toyota

**Affiliations:** 1 Institute of Symbiotic Science and Technology, Tokyo University of Agriculture and Technology2–24–16 Nakacho, Koganeishi, Tokyo 184–8588Japan

**Keywords:** ammonia-oxidizing archaea, ammonia-oxidizing bacteria, digestate, nitrification, soil microbial biomass

## Abstract

Wet and dry anaerobic fermentation processes are operated for biogas production from organic matter, resulting in wet and dry digestates as by-products, respectively. The application of these digestates to soil as fertilizer has increased in recent years. Therefore, we herein compared the effects of applying wet digestates (pH 8.2, C/N ratio 4.5), dry digestates (pH 8.8, C/N ratio 23.4), and a chemical fertilizer to Japanese paddy and upland soils on short-term nitrification under laboratory aerobic conditions. Chloroform-labile C, an indicator of microbial biomass, was only minimally affected by these applications, indicating that a small amount of labile N was immobilized by microbes. All applications led to rapid increases in NO_3_ -N contents in both soils, and ammonia-oxidizing bacteria, but not archaea may play a critical role in net nitrification in the amended soils. The net nitrification rates for both soils were the highest after the application of dry digestates, followed by wet digestates and then the chemical fertilizer in order of decreasing soil pH. These results suggest that the immediate effects of applying digestates, especially dry digestates with the highest pH, on nitrate leaching need to be considered when digestates are used as alternative fertilizers.

The soil nitrogen (N) balance is defined as the difference between the total quantity of N inputs for agricultural land and quantity of outputs (36). According to the Organization for Economic Cooperation and Development (OECD), Japan has the fourth-largest N balance among the 30 OECD member countries (28). Residual N in soil is mostly converted to nitrate and the leaching of nitrate from soil causes adverse environmental impacts such as eutrophication (12). Mishima *et al.* (24) reported the negative effects of a high N balance on the qualities of groundwater in Japan. The contribution of chemical fertilizers to N input is decreasing, whereas that of livestock manure is increasing in Japan (36). Therefore, nitrate leaching needs to be reduced following the application of organic matter including livestock manure to Japanese agricultural soils.

Biogas production from organic matter has increased in recent years, and, thus, the application of digestates, by-products of anaerobic digestion, to soil as biofertilizers has become more common (40). Two process types are applied to the production of biogas, and have been classified into wet and dry fermentation systems, with the most frequently applied type being the former (40). Wet digestion processes are operated for materials with total solid concentrations below 10%; therefore, wet digestates as by-products are pumpable and can be spread on fields for fertilization. Dry digestion processes are operated for materials with total solid concentrations between 15% and 35% and, hence, dry digestates have solid properties (40). Both digestates contain lower total organic carbon and higher NH_4_ -N contents and pH values than the original materials (10, 21, 25). Since the nitrification of ammonium to nitrate is one of the key processes determining nitrate leaching from soil, the nitrification processes of such digestates in soils need to be examined in order to predict potential environmental impacts. Strictly controlled laboratory experiments can provide important information on relative differences between these treatments (13).

The microbial immobilization of labile N after the application of organic matter to soil has been shown to reduce nitrification, and, thus, can decrease nitrate leaching (19, 21). Alburquerque *et al.* (2) reported that highly biodegradable digestates led to the immobilization of N and retarded nitrification, whereas less biodegradable digestates caused rapid nitrification. Therefore, the biodegradabilities of digestates need to be evaluated in order to more clearly understand nitrification processes in soil. However, few studies have investigated the biodegradability of dry digestates in soils, whereas wet digestates have been examined more extensively (2, 13).

Japanese agricultural land mainly consists of paddy and upland types. The nitrification processes of such digestates may also be influenced by differences between paddy and upland soils because nitrification can only occur in a thin surface layer and rhizosphere during rice growth in paddy soils covered with floodwater (4, 20).

The application of digestates may strongly influence nitrifying microbes. The oxidation of ammonium to nitrite is the rate limiting step in nitrification (22) and is catalyzed by ammonia monooxygenase (AMO), which is encoded by the *amoA* genes harbored by both ammonia-oxidizing archaea (AOA) and bacteria (AOB). However, the relative importance of AOA and AOB to the nitrification process is still under debate (17, 18). A real-time polymerase chain reaction (PCR) method has been developed to measure the copy numbers of genes. The use of such molecular methods has enabled the relative contribution of AOA and AOB to nitrification to be assessed. Saunders *et al.* (35) showed that the application of a digestate from wet fermentation only increased AOB, and not AOA *amoA* gene copies; however, the relationship between *amoA* gene copies and nitrification rates currently remains unclear.

The objective of this study was to compare the effects of the application of digestates and a chemical fertilizer to soils on short-term nitrification under laboratory aerobic conditions. We examined (1) the influence of the types of fermentation systems (*i.e.* wet and dry fermentation) on the rates of nitrification in paddy and upland soils and on microbial N immobilization, and (2) the relative contribution of AOA and AOB to nitrification following the application of digestates.

## Materials and Methods

### Soils and digestates used

Paddy and upland soils were collected from a 0–10 cm layer in spring in the Field Museum Hommachi, Field Science Center, Tokyo University of Agriculture and Technology, Fuchu, Tokyo, Japan. These soils are gray lowland soils (Fluvisols). Both the paddy and arable soils were not planted during winter. Paddy soil was collected before irrigation and was 42 g C kg^−1^ soil of total C, 4.3 g N kg^−1^ soil of total N, 364 mg C kg^−1^ soil of biomass C, 44 mg N ka^−1^ of biomass N, and 1 mg N kg^−1^ of NO_3_ -N at pH (H_2_ O) 5.5. The upland soil was 41 g C kg^−1^ soil of total C, 4.4 g N kg^−1^ soil of total N, 376 mg C kg^−1^ soil of biomass C, 50 mg N ka^−1^ of biomass N, and 41 mg N kg^−1^ of NO_3_ -N at pH (H_2_ O) 5.7. Biomass C and N were determined by a chloroform fumigation-extraction method (38) and were not significantly different between the paddy and arable soils. The NO_3_ -N content was significantly lower in paddy soil than in arable soil (*P*<0.001). Freshly collected soil samples were sieved to 2 mm and stored at field moisture at 5°C until use.

Two types of digestates from wet and dry anaerobic fermentation systems were used in the incubation experiments. The wet digestate was collected from a biogas plant in Aichi Prefecture, Japan, through wet fermentation from pig slurry. The dry digestate was collected from a biogas plant in Nagano Prefecture, Japan, through dry fermentation from kitchen waste and waste paper. We could not obtain the digestates from different fermentation systems with the same original materials because pig slurry contains less solid materials and is unsuitable for the dry fermentation process. Therefore, the different properties of the wet and dry digestates have been attributed to differences in the fermentation types and original materials in this study. Freshly collected digestates were stored at 5°C until use. The chemical properties of digestates are shown in Table 1. To estimate total C and N in digestates, digestates were firstly extracted using a 1:20 ratio of distilled water, and the remaining solid parts were then dried. Water soluble total C (WSC) and N of the extracts were measured with a TOC-V_CSH/CSN_ (Shimadzu, Kyoto, Japan). Water soluble NH_4_ -N was measured with the Indo-phenol blue method. Total C and N of solid parts were measured with a CN coder (MT-700, YANACO New Science, Kyoto, Japan). Total C and N were then estimated from the sum of water soluble and solid C and N. pH was determined in a 1:2 water-soluble extract.

### Soil incubation

**Fig. 1:**
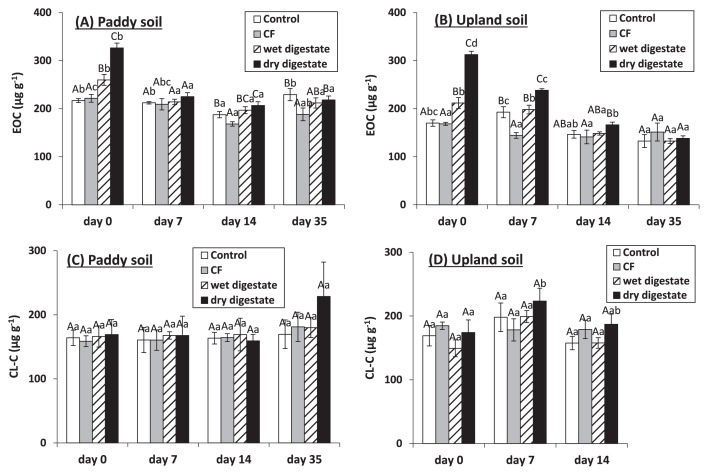
K_2_ SO_4_ -extractable organic C (EOC) (A and B) and chloroform-labile C (CL-C) (C and D) in paddy and upland soils after 0, 7, 14, and 35 d of incubation. CF indicates chemical fertilizer treatment. Bars indicate standard deviation (*n*=3). Different letters indicate significant differences between treatments (first uppercase letters) and sampling days (second lowercase letters) (*P*=0.05).

We arranged for soil to be subjected to 4 treatments in the incubation experiments, including the chemical fertilizer [8:8:8 N/P/K, Asahi Industries, Tokyo, Japan] (CF), the wet and dry digestate treatments, and a control (no addition). Amendments were added at an application rate of ~200 μg NH_4_ -N g^−1^ dry soil (equivalent to ~300 kg NH_4_ -N ha^−1^ assuming a soil bulk density of 1 g cm^−3^ up to a depth of 15 cm). The added amounts of wet and dry digestates were 58 and 75 mg g^−1^ dry soil (equivalent to 87 and 112 Mg ha^−1^), respectively. Using the NH_4_ -N content as an index of digestate application rates was a realistic approach since nitrogen is considered to be the main yield-limiting factor (1). Supplemented soil samples were mixed thoroughly with a spatula and weighed 5 g (oven-dried basis, 105°C) into 50 mL glass vials. The vials including soils were covered with aluminum foil and incubated for 0, 7, 14, or 35 d at 27°C. The moisture levels in the vials were maintained at 50% of the maximum water-holding capacity during the incubation with distilled water. K_2_ SO_4_ -extractable NO_3_ -N, organic C (EOC), total N (ETN), and pH, and chloroform-labile C (CL-C) and N (CL-N) as indicators of the soil microbial biomass, as well as AOA and AOB *amoA* gene copy numbers were analyzed using these vials, which were destructively collected with three replicates per treatment after 0, 7, 14, and 35 d of incubation (although we could not obtain the CL-C and CL-N values after 35 d of incubation for the upland soil because of a technical error [Fig. 1D]). pH (H_2_ O) was only measured after 0 d of incubation with no replication. Therefore, a total of 296 vials were prepared for two paddy and arable soils, four treatments (control and CF, wet digestate, and dry digestate applications), four sampling times (0, 7, 14, and 35 d), and three analyses (before and after chloroform fumigation and for DNA extraction) in triplicate, plus eight vials for the pH (H_2_ O) determination. Ammonia volatilization and denitrification were not measured because they were considered to be negligible due to homogeneous mixing of the digestates with soils and the incubation under aerobic conditions, as demonstrated by de la Fuente *et al.* (7).

### Analytical methods

Extractable NO_3_ -N, organic C (EOC), total N (ETN), and pH were determined following extraction using a 1:5 (w/v) ratio of soil to the 0.5 M K_2_ SO_4_ extract, similar to the approach by Galvez *et al.* (13). The concentration of NO_3_ -N was determined by the method of Cataldo *et al.* (3). EOC and ETN concentrations were measured with a TOC-V_CSH/CSN_ (Shimadzu) after sample filtration (0.45 μm pore diameter). CL-C and CL-N concentrations were determined as extractable C and N after chloroform fumigation subtracted from unfumigated (38), and were not converted to microbial biomass C and N because extractability by chloroform may be changed by the application of organic matter (32).

Soil DNA was extracted from 0.5-g soil subsamples taken from soil in the vials by the method of Sato *et al.* (34) and finally dissolved in 100 μL Tris-HCl, EDTA buffer. The abundances of the AOA and AOB *amoA* genes were quantified by real-time SYBR Green PCR assays in a Step One Real-Time PCR System (Life Technologies Japan, Tokyo, Japan). The primers used were *amoA*19IF (5′-ATGGTCTGGCTIAGACG-3′) (26) and *amoA*616R (5′-GCCATCCATCTGTATGTCCA-3′) (37) for the AOA *amoA* gene, and *amoA*-1F (5′-GGGGTTTCTACTGGTGGT-3′) and *amoA*-2R (5′-CCCCTCKGSAAAGCCTTCTTC-3′) for the AOB *amoA* gene (33). Each reaction was carried out in a 10-μL volume containing 2 μL of template DNA solution (diluted to 1/10), 5 μL of Fast SYBR Green I Master Mix (Life Technologies Japan), and 1.0 and 0.4 μmol L^−1^ of each primer for the AOA and AOB *amoA* genes, respectively. The cycling conditions were as follows: for the AOA *amoA* gene, an initial denaturation step at 95°C for 10 min followed by 40 cycles at 94°C for 30 s, 55°C for 30 s, and 72°C for 1 min (23); for the AOB *amoA* gene, an initial denaturation step at 94°C for 2 min followed by 45 cycles at 94°C for 30 s, 54°C for 30 s, and 72°C for 30 s (26). After each run, a melting curve was recorded between 60°C and 95°C to confirm the specificity of the real-time PCR assays. Standard curves were obtained using serial dilutions of linearized plasmids containing either a cloned AOA or AOB *amoA* gene. Data were linear for 10^0^ to 10^7^ gene copies. The amplification efficiencies of both genes were 80–84% and R^2^ values were > 0.998.

### Statistical analysis

All results are expressed as means and standard deviations on an oven-dried basis for three replicate measurements. The effects of the treatments and time of sampling on NO_3_ -N, EOC, ETN, CL-C, CL-N, and AOA and AOB *amoA* gene copies were analyzed by a two-way ANOVA followed by a Tukey mean comparison (*P*<0.05) using the software Excel Statistics version 12 (SPSS Japan, Tokyo, Japan).

## Results

### Digestates properties and soil pH changes

Marked differences were observed in the chemical properties of the wet digestate and dry digestate: the pH, total C, C/N ratio, and water soluble C were higher in the latter (Table 1). The higher total C, C/N ratio, and water soluble C in the dry digestate than in the wet digestate may have been due to the properties of original materials (*i.e.* kitchen waste and waste paper) with a high C/N ratio. In the present study, we did not establish why the dry digestate had a higher pH value.

The pH (H_2_ O) values after 0 d of incubation were 5.5, 5.7, 6.4, and 6.7 in the control, CF, wet digestate, and dry digestate treatments, respectively, for paddy soil, and 5.7, 5.5, 6.3, and 6.7 in the control, CF, wet digestate, and dry digestate treatments, respectively, for upland soil. The pH (H_2_ O) following application of the dry digestate was the highest among the treatments tested due to the high pH (H_2_ O) of the dry digestate (Table 1). pH in the K_2_ SO_4_ –soluble extract decreased until the first 7 d and ranged between 5.1 and 5.5 from 7 to 35 d following application of the wet and dry digestates. These values were similar to those for the control and CF treatments (data not shown).

### Changes in extractable and chloroform-labile C

Extractable organic C (EOC) at 0 d in both soils was significantly higher after the wet and dry digestate treatments than after the control and CF treatments (Fig. 1A and B). EOC at 0 d was significantly higher after the dry digestate treatment than after the wet digestate treatment due to the higher WSC in the dry digestate (Table 1). EOC rapidly decreased following the application of both digestates (Fig. 1A and B).

Chloroform-labile N (CL-N) can vary highly and sometimes have negative values (data not shown) because the CL-N measurement can be hampered by larger amounts of non-biomass N extracted from both fumigated and unfumigated soils due to the application of materials with high ETN contents (7). Therefore, chloroform-labile C (CL-C) was used as an indicator of the microbial biomass. While CL-C only significantly increased 7 d after the application of the dry digestate to upland soil, no other significant treatment or time effects were observed on CL-C (Fig. 1C and D).

### Changes in extractable NO_3_ -N and total N

**Fig. 2:**
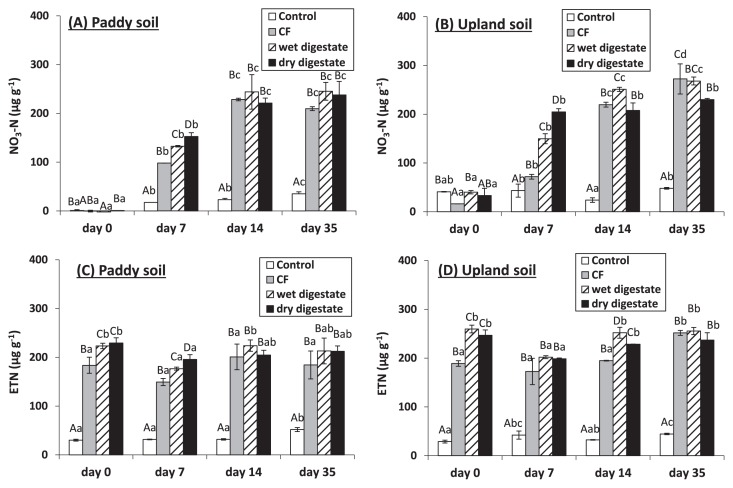
K_2_ SO_4_ -extractable NO_3_ -N (A and B) and total N (ETN) (C and D) in paddy and upland soils after 0, 7, 14, and 35 d of incubation. CF indicates the chemical fertilizer treatment. Bars indicate the standard deviation (*n*=3). Different letters indicate significant differences between treatments (first uppercase letters) and sampling days (second lowercase letters) (*P*=0.05).

NO_3_ -N at 0 d was significantly lower in control paddy soil than in control upland soil (*P*<0.001) (Fig. 2A and B). In the control treatments, NO_3_ -N significantly increased until 35 d in paddy soil, but appeared to remain constant in upland soil (Fig. 2A and B).

NO_3_ -N significantly increased until 14 d after N additions in both soils (Fig. 2A and B). NO_3_ -N at 7 d was significantly higher after the dry digestate application, following by the wet digestate and CF in both soils (Fig. 2A and B), indicating that the net nitrification rates were the highest after the dry digestate application. Changes in NO_3_ -N over time were similar in response to N additions between paddy and arable soils (Fig. 2A and B). NO_3_ -N remained unchanged and net nitrification was not observed from 14 to 35 d after application of the digestates because the nitrification rate may markedly decline with the rapid rates of initial nitrification.

Soil N additions resulted in increases in ETN, which remained at almost constant levels throughout the whole experiment period in both soils (Fig. 2C and D). ETN contents were almost similar to NO_3_ -N contents from 14 to 35 d of incubation in both soils (Fig. 2), indicating that the contribution of NH_4_ -N and labile organic N to ETN after 14 d was negligible.

### Changes in AOA and AOB amoA gene copy numbers

**Fig. 3:**
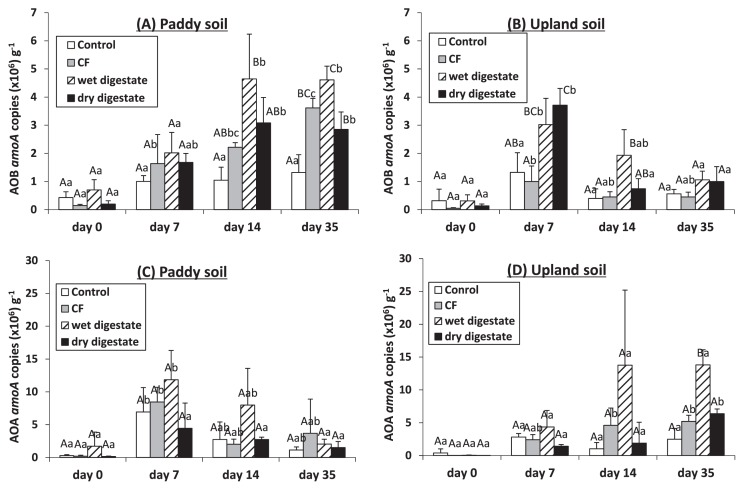
AOB *amoA* copies (A and B) and AOA *amoA* copies (C and D) in paddy and upland soils after 0, 7, 14, and 35 d of incubation. CF indicates the chemical fertilizer treatment. Bars indicate the standard deviation (*n*=3). Different letters indicate significant differences between treatments (first uppercase letters) and sampling days (second lowercase letters) (*P*=0.05).

In the control treatment, AOA *amoA* gene copy numbers significantly differed with time in paddy soil, but not in upland soil, whereas the AOB *amoA* gene copy numbers remained unchanged over 35 d in both soils (Fig. 3).

Soil N additions resulted in significant increases in AOB *amoA* gene copy numbers during 7 d of incubation in upland soil and during 14 d of incubation in paddy soil, and the growth of AOB was slightly faster in upland soil than in paddy soil (Fig. 3A and B). The growth of AOB was also slightly faster after the applications of the digestates than after that of CF (Fig. 3A and B). Although AOA *amoA* gene copy numbers increased slightly due to N additions, no significant differences were observed among treatments, except for the application of the wet digestate to upland soil on 35 d (Fig. 3C and D).

## Discussion

### Nitrification after the application of wet and dry digestates to paddy and upland soils

In the present study, both types of digestates increased soil extractable C (EOC) after 0 d of incubation (Fig. 1A and B) because they had water soluble C (Table 1). Since EOC represents the labile fraction of organic C that is easily used by microbes (13), the rapid decline observed in EOC over time may have been due to its consumption by microbes. However, CL-C did not increase after the application of the wet and dry digestates to both soils, with the exception of the dry digestate to upland soil after 7 d, although the concentrations of EOC decreased during the 7 d after the application of these digestates to both soils. Assuming that the ratio of respired to used EOC by microbes is 40% (30) and the extractability by chloroform fumigation is 0.45 (41), the increases observed in CL-C were estimated only below 27 μg C g^−1^ soil, which was difficult to detect as CL-C. Therefore, CL-C was only minimally affected by the application of the digestates used in this study. These results suggested that the immobilization of N by microbes was estimated only below 3.2 μg N g^−1^ soil assuming 60:7 as the C:N ratio in the microbial biomass (6) and that nitrification would occur readily without retarding due to the microbial immobilization of N. Rapid nitrification occurred after the application of the wet and dry digestates to both soils (Fig. 2A and B). C and N contents in the labile fraction of organic matter applied to soils were previously suggested to be better indicators for regulating N dynamics in the short-medium period than total C and N contents (13, 16, 21). Based on the C and N dynamics observed following the application of various types of digestates to soil, Alburquerque *et al.* (2) suggested that dissolved organic C < 5.5 g L^−1^ fresh weight (<45% of total organic C and <150% of total N) can be used to define less biodegradable digestates. Compared to these values, which were defined as less biodegradable, our digestates (even the dry digestate with a high C/N ratio) had markedly lower concentrations of water soluble C (WSC) (0.9 and 1.7 g C kg^−1^ fresh weight for wet and dry digestates, respectively) and proportions of WSC in total C and N (4.4% and 1.3% of total C and 20% and 30% of total N for wet and dry digestates, respectively) (Table 1), assuming that WSC was considered as dissolved organic C. Therefore, in the present study, the application of wet and dry digestates to both soils led to rapid nitrification without the lag phase associated with the immobilization of N by microbes (Fig. 2A and B). Previous studies reported that labile C contents in digestates were lower than those in raw materials because the labile fraction in total organic C was mostly consumed during anaerobic digestion, leading to the production of relatively stable (less biodegradable) digestates (5, 10, 14, 31). In addition, our results indicate that the degree of digestate stability may not depend on the fermentation processes (wet or dry).

Nitrate conversion (NC) was determined as the percentage of total N in the added digestate that had been converted into nitrate (2). NC during 35 d of incubation in this study was estimated as follows;

$$\text{NC }(\%)=100\times [{\left({\text{NO}}_{3}{\text{-N}}_{35\text{d}}-{\text{NO}}_{3}{\text{-N}}_{0\text{d}}\right)}_{\text{soil}+\text{digestate}}-{\left({\text{NO}}_{3}{\text{-N}}_{35\text{d}}-{\text{NO}}_{3}{\text{-N}}_{0\text{d}}\right)}_{\text{soil}}]/\text{added-N}.$$

NC was 82% and 49% for the wet and dry digestates, respectively, in paddy soil and 85% and 46% for the wet and dry digestates, respectively, in upland soil. These values were near to the relatively stable digestates (44–84%) reported by Alburquerque *et al.* (2), and suggested that the digestates used in this study were relatively stable materials. They proposed that relatively stable digestates with high NC values can be used directly in soils as good N-fertilizers because nitrate is the main form of N taken up by crops from the soil. However, the high application rates of these digestates to fields could lead to an increase in nitrate leaching, which may have negative impacts on the qualities of groundwater in Japan.

### Factors affecting nitrification

In the unamended control, nitrification only occurred in paddy soil (Fig. 2A and B). Fujii *et al.* (11) found a marked increase in the concentration of NO_3_ -N after plowing in spring in a Japanese paddy field, and attributed this finding to an increase in soil temperature and oxygen supply. We showed that the abundance of AOA, but not AOB *amoA* genes significantly increased over time in control paddy soil (Fig. 3). Fujii *et al.* (11) also observed an increase in the abundance of AOA *amoA* genes after plowing. This result in control paddy soil was also consistent with the findings that the growth of only AOA, but not AOB occurred in the control without N addition (9, 29, 44). This may have been because the growth of AOA was better at low ammonium concentrations (17).

In contrast, AOB responded to the application of CF as well as the wet and dry digestates to both soils, as indicated by the increase in AOB *amoA* gene copy numbers; however, AOA *amoA* gene copy numbers also increased slightly (Fig. 3). The net nitrification rates during the first 7 d of incubation (μg NO_3_ -N g^−1^ soil d^−1^) were calculated as follows;

$$\text{Net nitrification rate}=({\text{NO}}_{3}{\text{-N}}_{7\text{d}}-{\text{NO}}_{3}{\text{-N}}_{0\text{d}})/7.$$

**Fig. 4:**
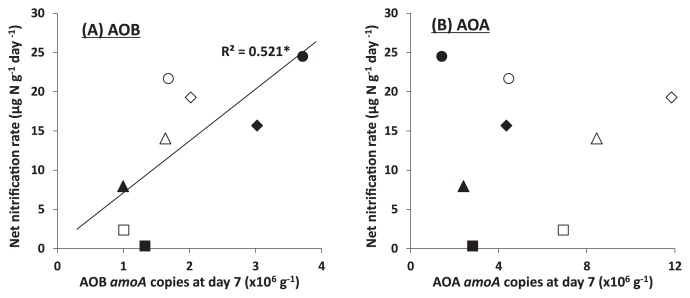
Relationships between net nitrification rates during 7 d and (a) AOB *amoA* copies and (b) AOA *amoA* copies after 7 d. Squares, triangles, diamonds, and circles denote control, chemical fertilizer, wet digestate, and dry digestate treatments, respectively. Open and closed symbols refer to paddy and arable soils, respectively.

The net nitrification rates only correlated with the AOB, but not the AOA *amoA* gene copy numbers on d 7 (Fig. 4). This result is consistent with the finding that AOB rather than AOA *amoA* gene copies correlated with nitrification after the addition of bovine urine to New Zealand soils (8, 9) and with potential nitrification activities in Japanese upland soils (26) and Chinese paddy soils (42). These results suggest that AOB may play a major role in nitrification after N additions to both soils because the growth of AOB may be favored at high ammonium concentrations receiving high N input (39).

**Fig. 5:**
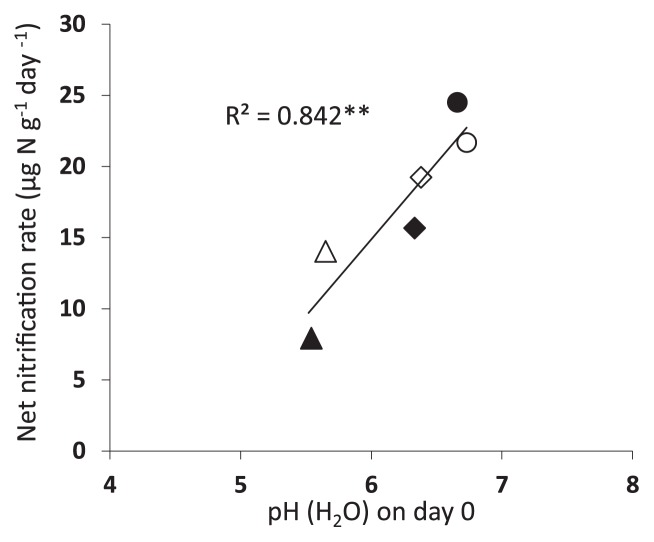
Relationship between net nitrification rates during 7 d and pH (H_2_ O) after 0 d. Triangles, diamonds, and circles denote chemical fertilizer, wet digestate, and dry digestate treatments, respectively. Open and closed symbols refer to paddy and arable soils, respectively.

Soil pH is a major factor affecting the activities of ammonia oxidizers that regulate soil nitrification because the available ammonia concentration is reduced by decreasing pH due to the ionization of ammonia (15). Nicol *et al.* (27) and Yao *et al.* (43) reported that the ratio of AOB to AOA *amoA* gene copies became higher with increasing soil pH because AOB adapts to environments with high ammonia concentrations. In the present study, we observed the highest net nitrification rates during the 7 d after the application of the dry digestate, which showed the highest pH values. We also found that the net nitrification rates after N additions correlated with soil pH (H_2_ O) (Fig. 5). These digestates are known to have generally higher pH values than original materials (10, 25). Therefore, the application of digestates to fields may increase the risk of nitrate leaching.

While the wet digestate can be used as both a basal dressing and top dressing because it can be spread on fields, the dry digestate can only be used as a basal dressing due to its solid properties. Therefore, the application of large amounts of the dry digestate to fields as a basal dressing and/or organic amendment, similar to manure, may increase the risk of nitrate leaching because the application of the dry digestate with a high pH value led to the rapid growth of AOB and, thus, rapid nitrification with less microbial N immobilization. Therefore, special attention is needed regarding the amounts and timing of the application of digestates, especially the dry digestate, in order to reduce nitrate leaching from soils when digestates are used as a substitution for chemical fertilizers and/or organic amendments.

## Figures and Tables

**Table 1:** Chemical properties of digestates used in the present study

	Water content (%)	pH (H_2_ O)	Total C (g C kg^−1^)	Total N (g N kg^−1^)	C/N ratio	WSC* (g C kg^−1^)	NH_4_ -N (g N kg^−1^)
Wet digestate	97	8.2	20.3	4.5	4.5	0.9	3.2
Dry digestate	77	8.8	131.2	5.6	23.4	1.7	2.6

Data are expressed on a fresh weight basis.

* WSC: water soluble C.
